# Non-invasive identification of swallows via deep learning in high resolution cervical auscultation recordings

**DOI:** 10.1038/s41598-020-65492-1

**Published:** 2020-05-26

**Authors:** Yassin Khalifa, James L. Coyle, Ervin Sejdić

**Affiliations:** 1 Department of Electrical and Computer Engineering, Swanson School of Engineering, University of Pittsburgh, Pittsburgh, PA USA; 2 Department of Communication Science and Disorders, School of Health and Rehabilitation Sciences, University of Pittsburgh, Pittsburgh, PA USA; 3 Department of Bioengineering, Swanson School of Engineering, University of Pittsburgh, Pittsburgh, PA USA; 4 Department of Biomedical Informatics, School of Medicine, University of Pittsburgh, Pittsburgh, PA USA; 5 Intelligent Systems Program, School of Computing and Information, University of Pittsburgh, Pittsburgh, PA USA

**Keywords:** Electrodiagnosis, Biomedical engineering

## Abstract

High resolution cervical auscultation is a very promising noninvasive method for dysphagia screening and aspiration detection, as it does not involve the use of harmful ionizing radiation approaches. Automatic extraction of swallowing events in cervical auscultation is a key step for swallowing analysis to be clinically effective. Using time-varying spectral estimation of swallowing signals and deep feed forward neural networks, we propose an automatic segmentation algorithm for swallowing accelerometry and sounds that works directly on the raw swallowing signals in an online fashion. The algorithm was validated qualitatively and quantitatively using the swallowing data collected from 248 patients, yielding over 3000 swallows manually labeled by experienced speech language pathologists. With a detection accuracy that exceeded 95%, the algorithm has shown superior performance in comparison to the existing algorithms and demonstrated its generalizability when tested over 76 completely unseen swallows from a different population. The proposed method is not only of great importance to any subsequent swallowing signal analysis steps, but also provides an evidence that such signals can capture the physiological signature of the swallowing process.

## Introduction

Electronic human activity monitoring devices and wearable technology have evolved in the past decade from simple macrodetection of gross events such as the number of steps taken during a walk around the block, to the detection of micro-events that exist within each gross event^1^. As a result, the quantity of data generated by these devices has exponentially increased along with the clinical questions arising with this data challenge^2^. Therefore, efforts to automate signal analysis are receiving more attention. Any systematic analysis of signals requires an important first step in which individual signal events are demarcated or segmented from one another before detailed analysis of signal components can be performed. This necessitates the development of robust automatic event detection methods to reduce the number of manual steps in signal analysis, mitigating human error and guaranteeing consistent detection criteria^3^. Event extraction algorithms have been introduced in many applications including speech analysis^4^, heart sounds segmentation^5^, brain signals analysis^6^, and swallowing activity analysis^3,7^. Many of these algorithms relied on multi-channel data to improve detection quality^8,9^.

All these applications share a common need of accurately defining the temporal borders (onset and offset) of certain events in order to be used for further processing and analysis. Particularly, we are interested in automated identification of vibratory and acoustic signals demarcating individual swallows using accelerometers and microphones^3^. Such automatic segmentation algorithms are critical for many applications that rely on swallowing sounds and vibrations which have been suggested as alternative bedside tools for dysphagia screening^10–18^, to discriminate between patients with healthy and dysphagic swallows^10,11^.

Dysphagia is a swallowing disorder that frequently follows stroke, neurodegenerative diseases, head and neck cancer and head injuries among many other etiologies^19^. Swallowing physiology and kinematics can be monitored and evaluated through various diagnostic imaging tools like endoscopy and ultrasound, but the gold standard is the videofluoroscopic swallowing study (VFSS). A typical VFSS is an X-ray procedure in which patients are asked to swallow different materials mixed with barium^20^. While VFSS is relatively efficient, its disadvantages include cost, short swallowing observation duration which fails to capture the variability of swallowing function occurring over the course of an entire meal, and limited availability to all clinicians and patients in no-acute care settings. It also has other disadvantages including radiation exposure and the need for specialized clinicians and equipment^19,21^. Even with institutional availability, VFSS cannot be used for daily and bedside assessment of swallowing^12^. These limitations increased interest in the use of noninvasive instrumental tools that help identify swallowing problems in the bedside and out of standard care settings.

Crude methods have been developed to use instrumentation for dysphagia screening through observing the patient’s behavior during swallowing. Instrumental screening acts as an initial evaluation that determines the necessity of performing more diagnostic exams such as VFSS. These methods include cervical auscultation which relies on a stethoscope to listen to the sounds emanating from the throat during swallowing in a similar way to listening to the sound of heart valves, blood flow, and airway. Experiments using cervical auscultation have reported subjectivity and low levels of inter-judge agreement when interpreting the sounds in addition to poor accuracy and reproducibility^22,23^. Conversely, high resolution devices which are independent of human auditory system, can record a wider spectrum of sounds and vibrations that the human auditory system is incapable of perceiving. High resolution cervical auscultation (HRCA) involves placing highly sensitive accelerometer and microphone to the anterior neck to capture swallowing vibrations and sounds in order to be objectively analyzed through advanced signal processing and machine learning algorithms. HRCA devices can capture multidimensional vibrations and inaudible components of swallowing sounds which with the appropriate analysis, can be superior to subjective acoustic analysis via stethoscope.

In recent years, acceleration and sound signals collected during swallowing have been the focus of many studies for the diagnosis and detection of dysphagia and its symptoms such as aspiration. These studies confirmed the presence of shared patterns among healthy swallows and the absence or delay of such patterns in dysphagic swallows^11,13,24–26^. Several studies used the sounds collected from surface microphones for aspiration detection and characterization of abnormal swallows through the analysis of power spectrum and distance based techniques^27,28^. The origin of swallowing vibrations picked through accelerometers has been investigated and correlated to hyolaryngeal excursion^14,29^ which paved the way for more studies that used swallowing accelerometry to evaluate airway protection^10,17,30,31^. However, most of these studies relied on expert manual segmentation of the swallowing signals by visual inspection of the concurrently collected diagnostic exams such as VFSS or repeated listening of sound signals.

Many swallowing event detection methods have been introduced in the literature especially for swallowing accelerometry. Sejdić *et al*.^3^ developed a segmentation algorithm that yielded over 90% accuracy for identifying individual segments for both simulated and real data. Their algorithm used sequential fuzzy partitioning of the acceleration signal based on its variance^3^. The output of partitioning from two orthogonal axes of acceleration (anterior-posterior and superior-inferior) was logically combined to achieve better detection of individual swallows and the algorithm was designed to deal with non-stationary long signals^3^. Damouras *et al*.^7^ proposed a volatility-based online swallow detection algorithm that works on raw acceleration signals. This algorithm achieved precision and recall values that are comparable to the results in^3^ and outperformed k-means and density-based spatial clustering of applications with noise (DBSCAN) algorithms^32^. Moreover, Lee *et al*.^12^, introduced a pseudo-automatic detection algorithm that depends on simple empirical thresholding of dual-axis accelerometry. They achieved high sensitivity, however the temporal accuracy of the detected segments was unacceptable compared to the expert manual segmentation. Other methods used manual segmentation either through inspection of acceleration by human experts^33^ or synchronizing with reference events in simultaneous videofluoroscopic studies^10,24^. Multi-sensor fusion was also used in swallowing segmentation by identifying the most useful signal combinations among three types of signals (dual-axis accelerometry, submental MMG, and nasal air-flow) achieving accuracies up to 89.6%^34^.

The purpose of this study is to evaluate the accuracy of spectral estimation and deep neural networks (DNNs) in automatic swallowing activity detection in both swallowing accelerometry signals and swallowing sounds. Three axes of acceleration and a single channel of swallowing sounds were investigated individually as standalone event detectors after which the best system was chosen according to detection quality when compared to the expert manual segmentation. Moreover, the used dataset overcomes the limitations of controlled data acquisition in the past segmentation studies, including number of subjects, swallowing maneuvers, swallowed materials and bolus size which represent most of the conditions common in dysphagia screening. This makes the dataset investigated in this study, optimal for the validation of such segmentation algorithm. We hypothesize that the proposed method will be able to correctly identify around 95% of the swallowing segment in more than 90% of attempts, irrespective of the texture or volume of the swallowed material, swallowing maneuver, or patient diagnosis.

## Results

A total of 3144 swallows (603 from stroke diagnosed patients and 2541 from other patients) were recorded with an average duration of 862.6 ± 277 msec. All the acquired signals (swallowing sounds and acceleration) from the microphone, and the three axes of the accelerometer were sampled at 20 kHz. Since numerous physiologic and kinematic events occur simultaneously during swallowing recordings (e.g. breathing, coughing), collected signals contain vibratory and acoustic information from multiple sources^7^. To overcome these and other measurement errors, we downsampled the entire dataset to 20% of the recorded sampling rate (i.e. 4 kHz instead of 20 kHz)^35^. All four signal streams (microphone, and accelerometer anterior-posterior [A-P], superior-inferior [S-I], and medial-lateral [M-L]) were independently considered for swallowing segmentation.

**Figure 1:**
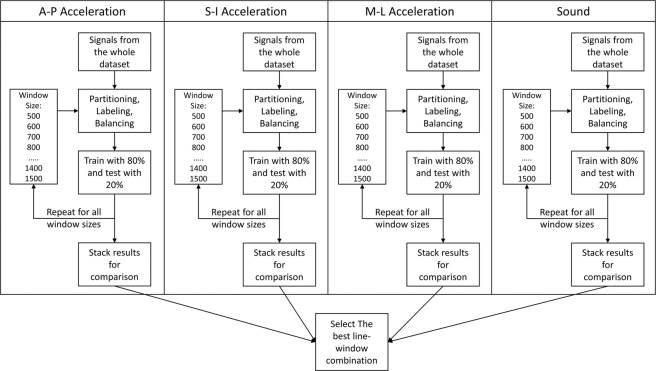
System’s parameter selection process.

To simulate the online processing scheme, and since we sought to determine whether automated segmentation could replicate gold-standard manual segmentation, a sliding window of size *N* samples was used to partition the signals into time samples. The window size *N* is considered as the predefined segmentation resolution of the system; therefore, we tested different values of *N* to see the effect of window size on the overall performance of the segmentation process. We used sizes of 500 to 1500 (25 *to* 375 *msec*) with a step of 100 samples and the selection of this range of values came from the fact that the acquired swallowing segments can be represented with the used window sizes. Moreover, a typical swallow segment can range in duration from 1 second (4k samples in this case) to more than 3 seconds which makes the selected window sizes robust to statistical error and efficient to detect the shortest swallows^7,36^. The algorithm was intended to use only non overlapping windows which reduces the number of processed windows and hence makes it suitable for real time operation; however, we considered a 50% overlap for all window sizes in order to test its effect on performance. So, four different segmentation models were trained and tested based on the four signal lines from microphone and accelerometer, each dependent on the spectrogram of underlying signal in order to determine the best window size and the best performing line as in Fig. 1.

**Figure 2:**
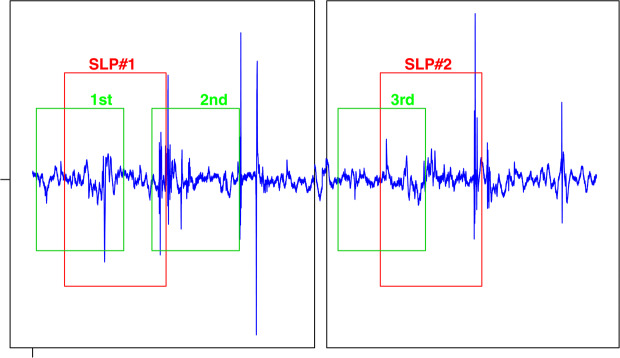
The labeling process of a sample swallow sound signal. Red windows represent the swallow segments identified by human expert SLP’s. Green windows represent different positions of the sliding window. The 1st and 3rd positions are labeled as swallows due to large overlap.

**Figure 3:**
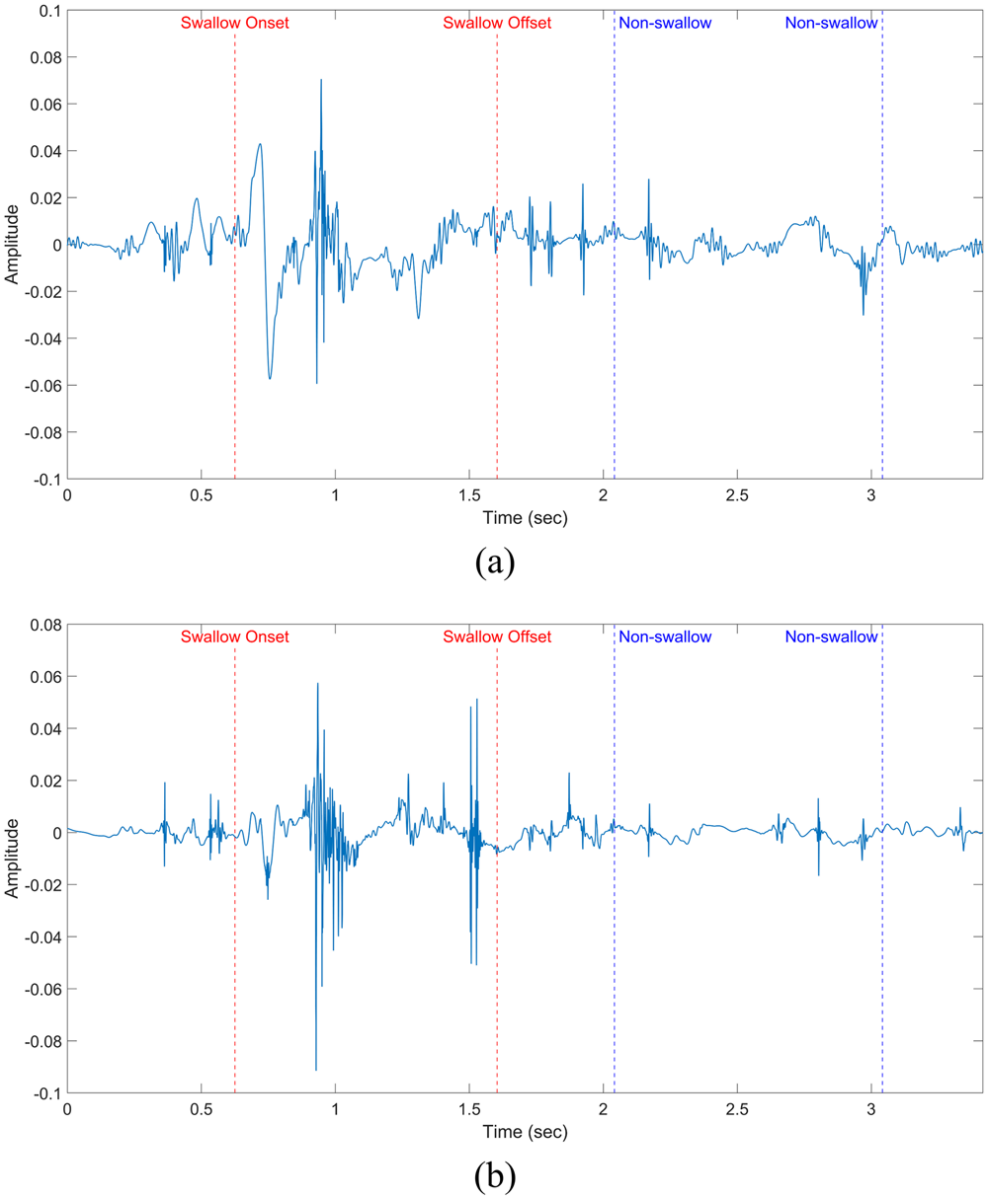
Sample raw sound and acceleration signals as recorded from the sensors attached on the anterior neck for each patient. The onset and offset of the swallow segment are marked in red dotted lines and the rest are non-swallow segments with the segment marked with the blue dotted lines as an example. (**a**) Microphone signal (**b**) A-P acceleration signal.

**Figure 4:**
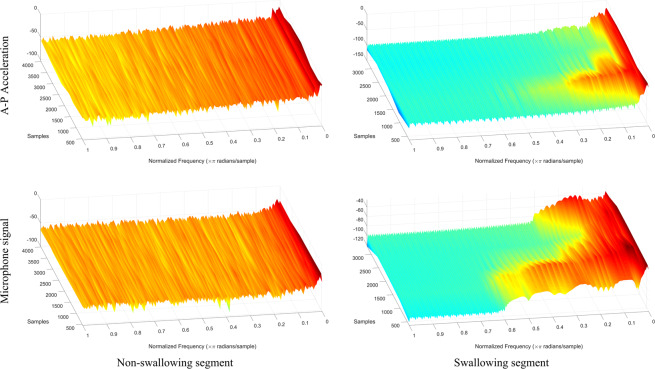
Spectrogram of non-swallow and swallow segments for both acceleration and sound.

All windows were labeled by comparing the start and end times to the timing of manual segmentation done by speech language pathologists (SLPs). A window is considered a part of a certain swallow if the the manually labeled swallowing segment overlaps with 50% or more of the automatically selected window size as shown in Fig. 2. The spectrogram of each window is calculated through the use of short-time Fourier transform (Eq. 1) with 5 non-overlapping time samples each of (*N*/5) length, a fixed length of 512 for the calculated Fourier transform and a Hanning window to reduce variance and leakage. This setup provided spectrograms of 257 frequency bins and we only used the magnitude of spectrogram in building the model while the phase was not of interest for this study. Fig. 3 shows sample signals as picked by the microphone and accelerometer with the onset and offset of the swallow segment marked with red dotted lines and an example of a non-swallow segment marked with blue dotted lines. Fig. 4 shows the spectrograms for the two segments (non-swallow and swallow) shown in Fig. 3 which basically represent the typical folded input into the DNN for each of the training models described previously. The magnitude of each spectrogram was unpacked into a (257 × 5) length vector to be used for the training process and prior training, all spectrograms were normalized to unit scale.

1 $$X(n,\omega )=\mathop{\sum }\limits_{m=-\infty }^{\infty }\,x[m]w[n-m]{\exp }^{-j\omega n}$$

The used window sizes produced 5574 to 20121 swallow windows and 9421 to 280043 non-swallow windows for window sizes 1500(375 *msec*) and 500(125 *msec*) respectively. This imbalance between swallows and non-swallows comes from the fact that each recording file contains longer blank (background noise) periods than swallowing periods. As a result, the balance between both types needed to be restored for the training of the system to mitigate bias. Therefore, we used the full set of the swallowing data at each window size and randomly selected an equal group of the non-swallowing data. Single swallows were also separated in order to form a smaller dataset so that we could test the system performance over single and other types of swallows (multiple and sequential) because the later categories are known to be more complex. The resultant datasets were randomly reordered and divided into two parts, 80% for training and 20% for testing.

A DNN was trained to create a feed forward probabilistic model of size 1285 × 1285 × 1 units. The DNN was created such that the input layer is the spectrogram vector of each window and the output layer represents the synthesized probability of whether the window is a part of a swallow or not. The output layer was configured to use the biased-sigmoid as an activation function with zero bias. The DNN was trained using a 100 iterations stochastic gradient descent (SGD)^37^. In addition, the DNN was configured to use dropout free training along with full sweep iterations of SGD.

**Figure 5:**
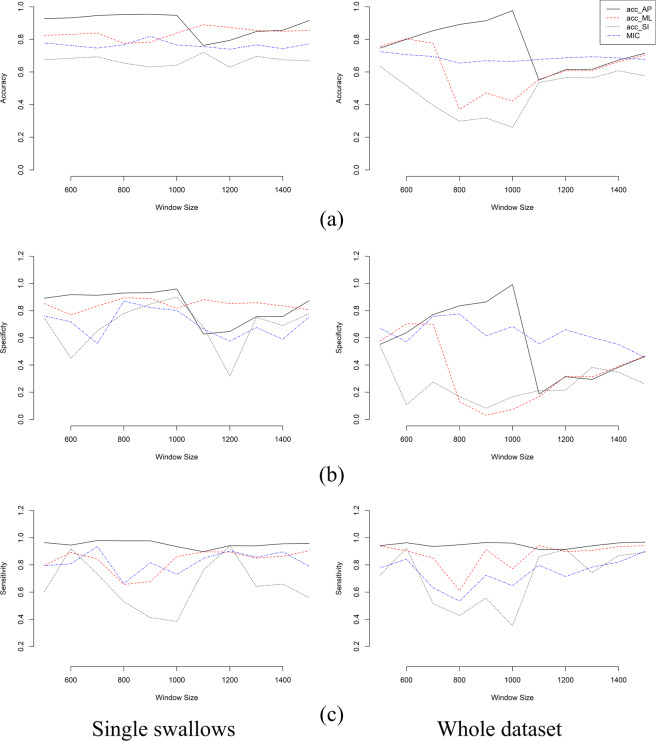
Quality measurements of the full run for the system. (**a**) Accuracy. (**b**) Specificity. (**c**) Sensitivity.

Fig. 5 shows the results of testing the DNN trained with 80% of the data for the three axes of accelerometer (A-P, S-I, and M-L) and microphone signal. At each window size, the performance of swallowing identification is shown in terms of accuracy, specificity, and sensitivity. According to Fig. 5, we can clearly see that the best results are achieved for A-P acceleration data at window sizes of 800 and 900 (900 and 1000 for the whole dataset). As a result, the 10-fold cross validation model was trained with A-P acceleration 10 times while excluding a randomly selected set of recordings each time for testing (without replacement). The top detection results achieved across all folds are shown in Table 1 for the two window sizes and the different overlap criteria. Ninety to 100% detection was accomplished for all four overlap ratios across single, multiple, and sequential swallows, and the precision of all four overlap ratios for single swallows was greater after post-processing. Multiple and sequential swallow detection also increased after post-processing however both the 900 and 1000 window sizes performed comparably. Overall, algorithm-based detection was most accurate using the window size of 800 (200 msec) for single swallows. On the other hand, using overlapping windows hasn’t had a noticeable effect on the algorithm performance except for long window sizes 1100–1500 (>225 msec). For A-P acceleration, the accuracy dropped between 1–5% for window sizes 500–1000 when using overlapping but increased with almost 8–12% for window sizes larger than 1100. Despite of the changes that overlapping induced to the performance, the best detection remains achievable at non-overlapping window sizes of 800–900.

**Table 1:** Detection measurements for the top two configurations.

Overlap Ratio	Property	Single Swallows		Multiple & Sequential Swallows	
		800 (200 *msec*)	900 (225 *msec*)	900 (225 *msec*)	1000 (250 *msec*)
2 SD below Average	Detected Swallows	100%	98.8%	96.5%	96.4%
	Average Duration (msec)	1461 ± 499.5	1564 ± 472.9	1335 ± 893.8	1474.1 ± 956.1
1 SD below Average	Detected Swallows	100%	97.7%	94.5%	95.3%
	Average Duration (msec)	1504.1 ± 465.7	1599.2 ± 452.3	1382.2 ± 631.6	1495.6 ± 644.2
90%	Detected Swallows	98.3%	90.8%	93.4%	94.2%
	Average Duration (msec)	1495.2 ± 355.9	1580.6 ± 270.4	1392 ± 625.8	1475.4 ± 366.5
95%	Detected Swallows	98.3%	90.8%	93.1%	94.2%
	Average Duration (msec)	1495.2 ± 355.9	1580.6 ± 270.4	1391.6 ± 625	1475.4 ± 366.5

**Figure 6:**
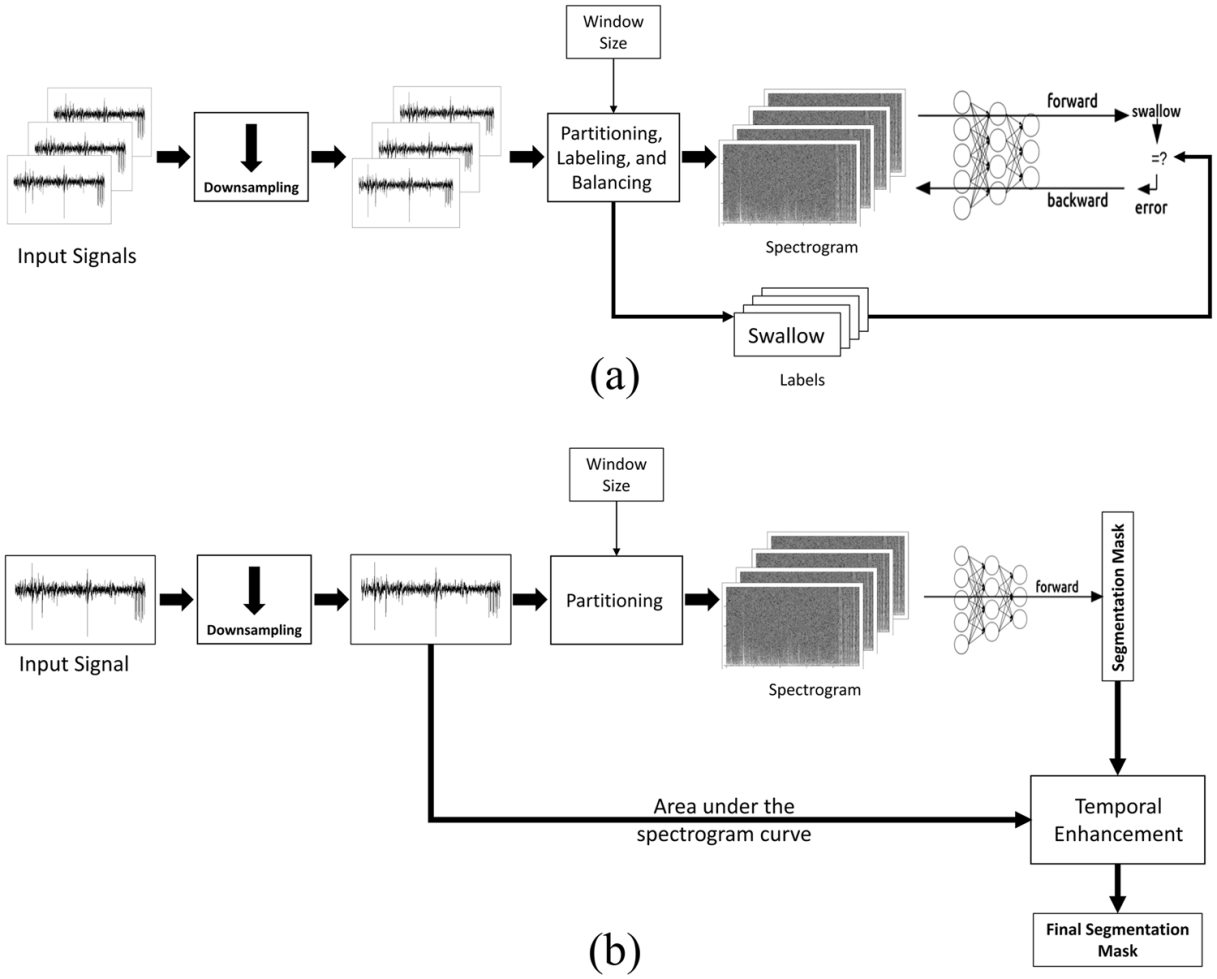
Flow of the training (**a**) and testing (**b**) paths of the proposed system.

**Figure 7:**
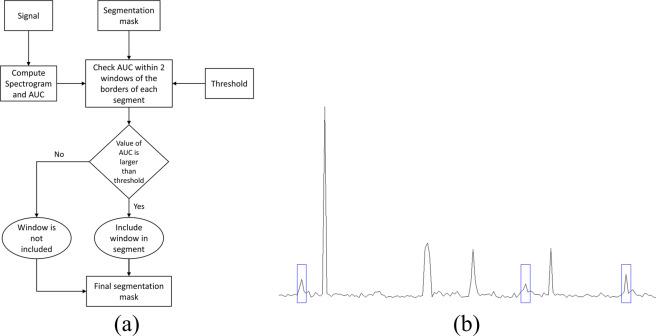
Temporal enhancement process: (**a**) shows the flowchart of the process. (**b**) A sample area under the spectrogram curve of a swallowing mic signal.

Once we got the best window size and the best performing line of swallowing signals from the parameter selection step, we retrained and tested the system using these parameters as the block diagram shows in Fig. 6. The whole dataset was divided randomly into 10 equal subsets in terms of recordings and a holdout method is repeated 10 times by training with 9 subsets and testing with the remaining one. Furthermore, the segmentation masks generated from this step were processed in order to enhance the temporal accuracy of the detection compared to the manual segmentation. This step is intended to check the boundaries of the detected segment and add a couple of samples on each side for a better match with SLP segments. The segments added to each side are determined through inspection of the area under the spectral estimate curve (AUC) of the swallowing signal (summation across frequencies for each time sample). The whole temporal enhancement process is illustrated in the flowchart shown in Fig. 7(a). The width of the segment is determined through simple thresholding of the AUC in the area around the detected segment with a threshold calculated from statistics of the segment (min and max). Figure 7(b) shows the AUC for a swallowing sound signal with swallowing segments annotated with rectangles. The inspection area was limited to 2 windows around the borders of each detected segment because more than this, will not be reasonable compared to the duration of swallows.

**Figure 8:**
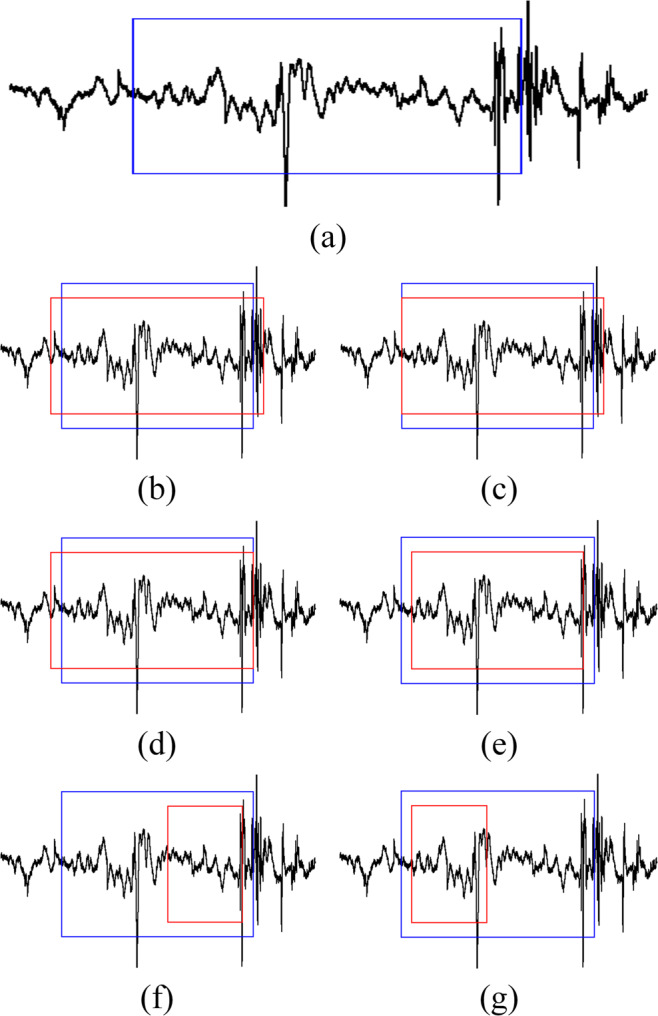
Possible swallow segmentation results. (**a**) Sample swallowing sound signal and definition of the swallowing segment (in blue). (**b**–**e**) Examples of correctly identified swallow segments (in red). (**f**,**g**) Examples of incorrectly identified swallow segments.

An assessment criterion was defined to validate the results of this segmentation work against the human expert manual segmentation as shown in Fig. 8. Manual segmentation defined swallow segments as the duration between the time when the leading edge of the bolus passes the shadow cast on the x-ray image by the posterior border of the ramus of the mandible (segment onset) and the time the hyoid bone completes motion associated with swallowing related pharyngeal activity and clearance of the bolus from the video image (segment offset). When patients swallow more than once to clear a single bolus (multiple swallow), the offset was based on the time when the hyoid returns to the lowest position before the next hyoid ascending movement associated with a subsequent swallow. A swallow segment was considered correctly identified (auto-detected) if and only if there exists a certain percentage overlap between the reference window determined by a human judge performing manual segmentation and the window produced by the proposed segmentation algorithm (as shown in Fig. 8(b–e))^3^. In this study, we tested multiple overlap ratios representing two different approaches. The first approach was a fixed overlap irrespective to the segment duration and the used overlap included 2 SD below the average swallow duration (431.89 *msec*)) and 1 SD below the average swallow duration (675.56 *msec*). The second approach was using a 90% and 95% overlap ratio of the manually measured duration for the compared segment. Otherwise the swallow was deemed to be incorrectly segmented (as shown in Fig. 8(f,g)). In addition to this assessment criterion, we used accuracy, specificity, and sensitivity to evaluate the overall performance of the segmentation process.

**Figure 9:**
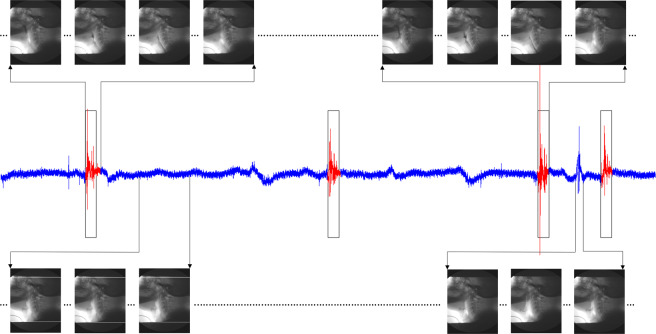
A clean A-P acceleration record. The red segments represent swallows as labeled by SLP. Black boxes are segments detected by the algorithm. Images on each corner are simultaneous VFSS snapshots of the signal events.

The algorithm also achieved 85.3 ± 12.5% sensitivity and 83.8 ± 9.5% specificity per each of the dataset records. These values were calculated over the whole dataset after removing the visually uncovered parts from records. The values came close to the anticipated results from the initial trials at Fig. 5. There may have been a slight drop in sensitivity and specificity due to misclassification at the borders of each swallow, in addition to the unlabeled swallows treated as false positives. These values go up to more than 90% for the clean records that don’t contain these pause areas and/or weren’t logged to have any visually missed events. Figure 9 shows the results of applying the segmentation algorithm on one of the clean records. It can be clearly seen that the algorithm successfully captured all the swallowing events in the signal and didn’t misidentify any part of the signal including the hyoid bone motion event prior to the last swallow (Fig. 9 lower right corner).

In order to further explore the performance of the proposed segmentation framework, it was evaluated as well in a standard clinical setup during the workflow of an ongoing swallowing experiment. A total of 76 swallows with an average duration of 1011 ± 216 msec, were used to test the proposed system for the detection of the onset and offset of pharyngeal swallows after being trained over the full 3144 swallows dataset mentioned previously. The used swallows in this validation procedure were meant to be completely unseen in order to test the robustness and generalizability of the proposed segmentation algorithm and never used in anyway in the training process. Both training and evaluation were performed using the best performing window size (800) and only the A-P acceleration. The segmentation framework presented interesting results when tested on the swallows from the independent clinical study where 97.4% of the swallows were correctly detected when considering an overlap window of 2 SD below the average swallow duration calculated from the original dataset, 84.2% of the swallows for 1 SD below average swallow duration, and 65.8% of the swallows when considering overlap ratios of 90% or more.

The videofluoroscopy instrument was controlled by a radiologist who had a switch to stop the imaging procedure when there was no bolus administered to the patient in order to reduce the radiation dose. This pausing in the x-ray machine operation caused the collected videos to have static frames for long periods with no visual clue about the events occurring while vibratory and acoustic data continued to be recorded. These events included swallows, talking, coughing, and head motion occuring between elicited swallow events. Without the visual help of VFSS, these events cannot be labeled; hence, included in the evaluation of this segmentation procedure. However, the algorithm was applied to these areas after training to see if it would pick up any of these events. This, alongside with the presence of unexplained false positives, necessitated manual inspection and validation of the segmentation results against the videos and logs kept by research associates collecting the swallow data. A trained rater validated each event detected by the algorithm in order to identify the origin of non-swallow events as a qualitative assessment for the proposed algorithm. More than 6500 detected segments were analyzed and validated visually against the videos and session logs for a window size of 900 and a 90% overlap criterion. The outcomes of the analysis (Table 2) show that the algorithm captured more than 94% of the swallows which is nearly a match with the results of the whole dataset in Table 1. Moreover, the rater reported that the algorithm successfully detected 353 swallows that were not captured/labeled in videos. The visually uncovered events reported in Table 2, are the segments detected by the algorithm during video pause times with no reference in session logs.

**Table 2:** Outcomes of the manual validation of automatic segmentation results.

Event type	Details		Total count
Swallowing events	Detected by the algorithm		2225
	Undetected by the algorithm	128	2353
Non-swallowing events	Reduced oral containment (Premature Spillage)	38	1275
	Hyoid bone movement	434	
	Coughing	134	
	Head and neck movement	266	
	Unexplained	403	
Visually uncovered events			2936

## Discussion

The results confirmed our hypothesis that the proposed algorithm can correctly and without human intervention, detect 95% of known swallow durations in more than 90% of attempts across simple (clean swallows) and complex (non-swallow activity co-occurring with swallows) swallow events. We can clearly see from Fig. 5 that training a DNN with the spectral estimate for the raw swallowing vibrations of a single channel can produce accuracies as low as 26.1% and up to 97.6% on window level over the whole dataset. In addition, the system showed robustness in terms of true positive and true negative rates. The best performing channel was the A-P accelerometer axis with an average accuracy of 89.44% for single swallows (75.9% for the whole dataset) and superior sensitivity and specificity which is comparable to the results in^7^. The performance of other channels was close to the A-P axis, but the lowest performance was given by feeding the network with the spectrogram of the SI axis for all considered quality measurements.

The selection of proper window size highly depends on signal temporal characteristics which is obviously clear in the demonstrated results. We stated that the whole set of collected swallows is on average of 862.6 ± 277 msec. This makes the best window size to detect these swallows, located around the middle of used range (800–1000) because each swallow can be represented as integer multiples of the selected window in this range especially since we did not use any overlap between the sliding windows. This effect is most highly illustrated in the results of the A-P acceleration where the accuracy, true negative and true positive rates increase to their maximum at window size of (900–1000) and then drop sharply. They return to increase after this drop because the window size increases and approaches multiples of the effective values mentioned before. The effect is almost the same with other components of acceleration and microphone signals.

The temporal accuracy of detection was examined as well for the best two systems with window sizes of 800 and 900 for single swallows (900 and 1000 for the whole dataset) and validated against the manual segmentation by SLPs as shown in Table 1. Among the examined assessment criteria, we found that a 2 SD below average swallow duration criterion as the minimum overlap (431.89 *msec* ≈47% of average swallow duration) between the detected and manually segmented swallows, is very low considering the duration of the examined swallows, however it gives excellent detection results. So, we tested 1 SD below the average duration (675.56 *msec* ≈73.5% of average swallow duration) as well as 90% and 95% minimum overlap. The average duration of detected segments in the three criteria are close in duration and all of them are not far from the average duration of the actual segments. Moreover, the fluctuations in segment duration are considered very convenient compared to the length of segments. Therefore, all of these criteria proved to deliver excellent automated detection accuracy of swallow events without human intervention.

Encouragingly, the system has also shown promising segmentation quality when applied on completely unseen data collected from different group participants with control parameters that were not included in the main dataset under investigation. Despite these promising results, there is a little drop in the number of swallows correctly segmented considering different overlap windows when compared to the original dataset. The reason behind this drop in performance may be returned to the fact that there is actually a difference in the average swallow duration between the two datasets (a little longer in case of swallows from healthy participants) which in turn reflects on the needed window size that best represents the swallows. Another factor that may contributed into this performance drop, is the possibility that the used set of swallows contains some multiple swallows which causes the detection quality to drop when included as shown in Table 1. Nevertheless, the performance presented by the system on the new dataset suggests that it is likely to generalize to other swallowing datasets.

Evidently, the proposed algorithm achieved results better than most of the swallowing signal segmentation algorithms in the literature, especially the work in^3^ which achieved the best swallowing segmentation accuracy in swallowing accelerometry. In this work, Sejdić *et al*.^3^ performed a maximum likelihood estimation to calculate the onset and offset times of swallows in acceleration signals. They used also a good dataset with multiple swallowing maneuvers and materials; however, the algorithm is computationally expensive especially when the number of swallowing segments in the signal is unknown. Damouras *et al*.^7^ used quadratic variation that is extracted directly from acceleration signals to perform segmentation and the algorithm was computationally effective to execute. Their algorithm achieved recall values up to 94% but it was highly affected by the presence of noise and the used dataset wasn’t diverse enough considering the maneuvers and material consistencies. The work of Lee *et al*.^34^ also achieved good segmentation quality (accuracy up to 89.6%) through the use of sensor fusion and neural networks but they didn’t provide any analysis to show the detection quality on the temporal side of the swallowing segments and the reference manual segmentation used was done for swallowing apnea which is shorter than the swallow itself. On the other hand, our proposed algorithm is validated using a wide dataset rather than a controlled limited dataset like most of the previous studies. The used dataset is at least 10 times larger than any used dataset in swallowing segmentation and covered most of the known swallowing conditions encountered in dysphagia screening which occurs in typical healthcare environments that allow for very limited control of patient position and other variables. This is important because our results, obtained in a naturalistic setting, are more externally valid than they would be had the data been collected under strict experimental controls as seen in many prior published studies. In addition, the proposed algorithm has a better response time in testing phase that doesn’t exceed milliseconds and is suitable for real time processing and use on edge mobile devices. The algorithm uses massive computational resources for the training phase like any deep neural network, but this can be overcome using the newly emerging platforms with GPUs or special architectures to accelerate the training process. The use of deep neural networks along with the time-frequency representation of swallowing signals was able to model the fine differences between swallowing segments and other events captured given the power of neural networks in efficient feature and parameter learning procedures. The future work for the proposed algorithm will include fusion between different signal lines in order to achieve more robust segmentation and avoid the detection of false positive events such as coughing and head movement. We will include also recurrent neural networks for their power in modeling long range dependencies in time series in addition to functionally employing longer window sizes and overlapping to guarantee better detection quality especially at the borders of the swallow (onset and offset).

The start and end of each pharyngeal swallow can be roughly identified through visual and tactile inspection of hyo-laryngeal excursion and other observations of the patient swallowing. However, these methods are subjective and not reliable. Traditional cervical auscultation using a stethoscope to observe swallowing sounds, is particularly unreliable despite its commonplace use. This renders the advancements in high resolution cervical auscultation and machine learning methods demonstrated in this investigation and others, especially encouraging toward a goal of unsupervised detection of swallow events and many of their physiologic components and more timely identification of patients with dysphagia who need intervention. Adding a robust method that can automatically identify swallows is of a great clinical significance to diagnosis and rehabilitation of swallowing disorders. Such methods can detect swallows that are hard to observe in patients who have difficulty initiating oropharyngeal swallow (e.g. Parkinson’s disease) or patients with weak pharyngeal swallow (e.g. medullary stroke)^38^. Future directions for this technology include developing computational deglutition methods to pre-emptively detect airway compromise (e.g. aspiration) and other clinically significant swallowing disorders at the bedside^30^, facilitate behavioral treatments by providing real-time swallow biofeedback^19^, and in day-to-day management of swallowing disorders in settings that lack adequate qualified dysphagia clinical specialists.

## Conclusion

In this paper, a novel automatic segmentation algorithm for swallowing accelerometry and sounds was proposed, and its potential in dysphagia screening was discussed. The algorithm scans the swallowing signals through a sliding window of a specific size and each window is classified as a swallow or non-swallow through feeding its spectral estimate into a deep neural network. Swallowing signals from 248 participants were collected for different swallowing tasks, manually labeled by experts and used to train and validate the system. The proposed algorithm yielded over 95% accuracy at the window level in addition to similar values of sensitivity and specificity. On the temporal side, the algorithm nearly did not fail in detecting any swallowing activity (2 SD below average) and proved superior in detection despite high overlap ratios with accuracies that exceeded 90% for all types of swallows. Moreover, the algorithm showed similar performance when tested on completely unseen data implying the ability to generalize to other datasets. Our algorithm has demonstrated the potential of deep neural networks and spectral representation of swallowing signals to event detection in swallowing accelerometry.

## Methods

**Figure 10:**
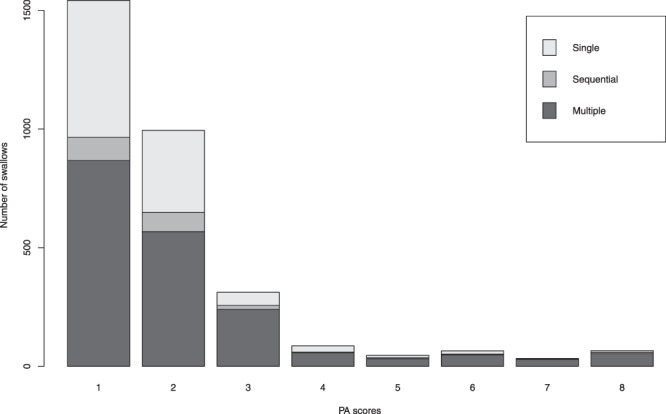
Number of swallows in the dataset for each PA score.

This study was approved by the Institutional Review Board of the University of Pittsburgh. All participating patients gave informed consent to join the study. All experiments were performed in accordance with relevant guidelines and regulations. A total of 248 patients (148 males, 100 females, age: 63.8 ± 13.7) served as the sample for this experiment. They were recruited from the population of patients referred to the Speech Language Pathology service for an oropharyngeal swallowing function assessment with videofluoroscopy at the University of Pittsburgh Medical Center (Pittsburgh, PA), due to clinical suspicion of dysphagia. Of the sample, 44 patients (32 males, 12 females, age: 66.6 ± 13.7) were diagnosed with stroke while the remaining 204 patients (116 males, 88 females, age: 63.0 ± 14.3) had medical conditions unrelated to stroke. Patients were asked to swallow multiple materials of different viscosities and volumes including chilled (5 °*C*) Varibar thin liquid (Bracco Diagnostics Inc., Monroe Township, NJ), chilled (5 °*C*) Varibar nectar, honey thick liquid, barium tablets (EZ Disk, Bracco Diagnostics Inc., Monroe Township, NJ), Varibar pudding, or a cookie coated with Varibar pudding. The swallows were performed with and without verbal command and in multiple maneuvers including neutral, chin down, left and right head rotation, combined chin down and head rotation, Supraglottic swallow (SGS), and modified SGS. The vibrations of each swallow were recorded as a separate task by the LabView Signal Express and exported in a plain text format to be used for subsequent analysis. A total of 3144 swallows (603 from stroke diagnosed patients and 2541 from other patients) were recorded with an average duration of 862.6 ± 277 msec. The collected swallows included 1038 single swallows, 1893 multiple swallows (several swallows to swallow a single bolus) and 213 sequential swallows (swallows of more than one bolus one at a time in a rapid sequence). The whole set of collected swallows, was used entirely to train and evaluate the proposed segmentation framework regardless of the consistency of the swallowed material and/or the administered bolus volume. This assures that the collected dataset covers as many as possible of the swallowing conditions common in day-to-day swallowing assessment and that the proposed segmentation framework will be trained and evaluated across a diverse rather than controlled dataset which guarantees robustness and adaptability to deployment in standard clinical care conditions. The swallowing event start (onset) and end (offset) times taken as gold standard for the experiment were obtained through manual segmentation of videofluoroscopy sequences by experienced SLPs in our Swallowing Research Lab along with the penetration aspiration (PA) scores^39^ of the swallows as described in^40^. PA scale scores indicate the depth of entry of swallowed material into the patient’s airway when swallowing, and the quality of the patient’s airway protective response to airway penetration (material remaining above the true vocal folds) or aspiration (material coursing through the larynx and entering the trachea). The number and type of swallows in each PA score are summarized in Fig. 10.

Data acquisition was performed per previous work published by Dudik *et al*.^41^. The swallowing vibrations were recorded during a routine videofluoroscopy with two types of sensors, a tri-axial accelerometer (ADXL 327, Analog Devices, Norwood, Massachusetts) and a lapel microphone (model C 411L, AKG, Vienna, Austria) attached to the subject’s anterior neck. The accelerometer complex (sensor in a plastic case) was attached to the skin overlying the cricoid cartilage for the best signal quality^42^. The first two axes of accelerometer were aligned to the anterior-posterior (A-P) and superior-inferior (S-I) directions which can be described as perpendicular to the coronal plane and parallel to the cervical spine respectively. The third axis of accelerometer (medial lateral axis or M-L) was parallel to the axial/transverse plane of the patient’s head and neck. The sensor was powered using a 3V power supply (model 1504, BK Precision, Yorba Linda, California) and had its output signals hardware band-limited to 0.1–3000 Hz and amplified with a gain of 10 (model P55, Grass Technologies, Warwick, Rhode Island).

The microphone was mounted towards the right lateral side of the larynx with no contact with the accelerometer to avoid any friction noise and to avoid obstructing the upper airway radiographic view, and powered via a microphone specific power supply (model B29L, AKG, Vienna, Austria) with the maximum possible volume level (9 for this device). The conditioned signals from the microphone and accelerometer were fed into a National Instruments 6210 DAQ, sampled at a 20 kHz rate, and acquired by LabView’s Signal Express (National Instruments, Austin, Texas). The previous setup for both accelerometer and microphone has proven to be effective in collecting swallowing vibrations^42–45^. A video capture card (AccuStream Express HD, Foresight Imaging, Chelmsford, MA) was used to feed the output of the videofluoroscopy instrument (Ultimax system, Toshiba, Tustin, CA) into LabView for recording. All signals fed to the DAQ were acquired and recorded simultaneously for a complete start-to-end synchronization.

An identical collection procedure to the aforementioned one, was used for the clinical experiment that yielded the swallows used for testing the generalizability of the proposed system. The experiment was performed on healthy community-dwelling adults who had no history of swallowing difficulties. Twenty subjects (9 males, 11 females, age: 65.8 ± 11.4) who provided informed consents, participated in the experiment. The participants in this sample were selected randomly from a population that had no history of surgeries to the head or neck region or neurological disorders and underwent swallowing evaluation as a part of bigger study. Only thin liquid boluses: 3 mL by spoon and unmeasured self-administered volume cup sips, were administered to the subjects in a completely randomized order.

## Data Availability

The dataset used in this study might be available upon reasonable request from the corresponding author and with permission from our clinical collaborators.

## References

[1] Rashidi P, Mihailidis A (2013). A survey on ambient-assisted living tools for older adults. IEEE Journal of Biomedical and Health Informatics.

[2] Andreu-Perez J, Poon CCY, Merrifield RD, Wong STC, Yang GZ (2015). Big data for health. IEEE Journal of Biomedical and Health Informatics.

[3] Sejdić E, Steele CM, Chau T (2009). Segmentation of dual-axis swallowing accelerometry signals in healthy subjects with analysis of anthropometric effects on duration of swallowing activities. IEEE Transactions on Biomedical Engineering.

[4] Park SS, Kim NS (2007). On using multiple models for automatic speech segmentation. IEEE Transactions on Audio, Speech, and Language Processing.

[5] Huiying, L., Sakari, L. & Iiro, H. A heart sound segmentation algorithm using wavelet decomposition and reconstruction. In *Engineering in Medicine and Biology Society, 1997. Proceedings of the 19th Annual International Conference of the IEEE*, vol. 4, 1630–1633 vol.4 (1997).

[6] Lan, T., Erdogmus, D., Pavel, M. & Mathan, S. Automatic frequency bands segmentation using statistical similarity for power spectrum density based brain computer interfaces. In *Proceedings of the International Joint Conference on Neural Networks, IJCNN 2006, part of the IEEE World Congress on Computational Intelligence, WCCI 2006, Vancouver, BC, Canada, 16*–*21 July 2006*, 4650–4655 (2006).

[7] Damouras S, Sejdić E, Steele CM, Chau T (2010). An online swallow detection algorithm based on the quadratic variation of dual-axis accelerometry. IEEE Transactions on Signal Processing.

[8] Lehner RJ, Rangayyan RM (1987). A three-channel microcomputer system for segmentation and characterization of the phonocardiogram. IEEE Transactions on Biomedical Engineering BME.

[9] Chan HL, Lin CH, Ko YL (2003). Segmentation of heart rate variability in different physical activities. Computers in Cardiology.

[10] Lee J (2006). A radial basis classifier for the automatic detection of aspiration in children with dysphagia. Journal of NeuroEngineering and Rehabilitation.

[11] Reddy NP, Thomas R, Canilang EP, Casterline J (1994). Toward classification of dysphagic patients using biomechanical measurements. J Rehabil Res Dev.

[12] Lee J, Steele CM, Chau T (2008). Time and time–frequency characterization of dual-axis swallowing accelerometry signals. Physiological Measurement.

[13] Reddy N (1991). Noninvasive acceleration measurements to characterize the pharyngeal phase of swallowing. Journal of Biomedical Engineering.

[14] Reddy NP (2000). Measurements of acceleration during videofluorographic evaluation of dysphagic patients. Medical Engineering and Physics.

[15] Rebrion C (2019). High-resolution cervical auscultation signal features reflect vertical and horizontal displacements of the hyoid bone during swallowing. IEEE Journal of Translational Engineering in Health and Medicine.

[16] He Q (2019). The association of high resolution cervical auscultation signal features with hyoid bone displacement during swallowing. IEEE Transactions on Neural Systems and Rehabilitation Engineering.

[17] Yu, C., Khalifa, Y. & Sejdic, E. Silent aspiration detection in high resolution cervical auscultations. In *2019 IEEE EMBS International Conference on Biomedical Health Informatics (BHI)*, 1–4 (2019).

[18] Mao S (2019). Neck sensor-supported hyoid bone movement tracking during swallowing. Royal Society Open Science.

[19] Reddy NP (2000). Biofeedback therapy using accelerometry for treating dysphagic patients with poor laryngeal elevation: case studies. Journal of Rehabilitation Research & Development.

[20] Mohammadi H, Steele C, Chau T (2016). Post-segmentation swallowing accelerometry signal trimming and false positive reduction. IEEE Signal Processing Letters.

[21] Dudik JM, Coyle JL, Sejdić E (2015). Dysphagia screening: Contributions of cervical auscultation signals and modern signal-processing techniques. IEEE Transactions on Human-Machine Systems.

[22] Zenner PM, Losinski DS, Mills RH (1995). Using cervical auscultation in the clinical dysphagia examination in long-term care. Dysphagia.

[23] Leslie P, Drinnan MJ, Finn P, Ford GA, Wilson JA (2004). Reliability and validity of cervical auscultation: A controlled comparison using video fluoroscopy. Dysphagia.

[24] Chau T, Chau D, Casas M, Berall G, Kenny DJ (2005). Investigating the stationarity of paediatric aspiration signals. IEEE Transactions on Neural Systems and Rehabilitation Engineering.

[25] Das A, Reddy NP, Narayanan J (2001). Hybrid fuzzy logic committee neural networks for recognition of swallow acceleration signals. Computer Methods and Programs in Biomedicine.

[26] Reddy NP, Costarella BR, Grotz RC, Canilang EP (1990). Biomechanical measurements to characterize the oral phase of dysphagia. IEEE Transactions on Biomedical Engineering.

[27] Shirazi SS, Buchel C, Daun R, Lenton L, Moussavi Z (2012). Detection of swallows with silent aspiration using swallowing and breath sound analysis. Medical & biological engineering & computing.

[28] Lazareck LJ, Moussavi ZMK (2004). Classification of normal and dysphagic swallows by acoustical means. IEEE Transactions on Biomedical Engineering.

[29] Zoratto DCB, Chau T, Steele CM (2010). Hyolaryngeal excursion as the physiological source of swallowing accelerometry signals. Physiological Measurement.

[30] Sejdić E, Steele CM, Chau T (2013). Classification of penetration–aspiration versus healthy swallows using dual-axis swallowing accelerometry signals in dysphagic subjects. IEEE Transactions on Biomedical Engineering.

[31] Steele CM, Sejdić E, Chau T (2013). Noninvasive detection of thin-liquid aspiration using dual-axis swallowing accelerometry. Dysphagia.

[32] Dudik JM, Kurosu A, Coyle JL, Sejdić E (2015). A comparative analysis of DBSCAN, K-means, and quadratic variation algorithms for automatic identification of swallows from swallowing accelerometry signals. Comput. Biol. Med..

[33] Hanna F, Molfenter SM, Cliffe RE, Chau T, Steele CM (2010). Anthropometric and demographic correlates of dual-axis swallowing accelerometry signal characteristics: A canonical correlation analysis. Dysphagia.

[34] Lee J, Steele CM, Chau T (2009). Swallow segmentation with artificial neural networks and multi-sensor fusion. Medical Engineering & Physics.

[35] Russell, J. R. & Bandi, F. M. Microstructure noise, realized volatility, and optimal sampling. Econometric Society 2004 Latin American Meetings 220, Econometric Society (2004).

[36] Sonies BC, Parent LJ, Morrish K, Baum BJ (1988). Durational aspects of the oral-pharyngeal phase of swallow in normal adults. Dysphagia.

[37] Simpson, A. J., Roma, G. & Plumbley, M. D. Deep karaoke: Extracting vocals from musical mixtures using a convolutional deep neural network. In *International Conference on Latent Variable Analysis and Signal Separation (LVA/ICA)*, 429–436 (2015).

[38] Logemann, J. A. *Evaluation and treatment of swallowing disorders* (Austin, Tex.: PRO-ED, c1983, 1998).

[39] Rosenbek, J. C., Robbins, J. A., Roecker, E. B., Coyle, J. L. & Wood, J. L. A penetration-aspiration scale. *Dysphagia* **11**(2), 93–98 (1996). 10.1007/BF00417897 8721066

[40] Robbins J, Coyle J, Rosenbek J, Roecker E, Wood J (1999). Differentiation of normal and abnormal airway protection during swallowing using the penetration–aspiration scale. Dysphagia.

[41] Dudik JM, Kurosu A, Coyle JL, Sejdić E (2016). A statistical analysis of cervical auscultation signals from adults with unsafe airway protection. Journal of neuroengineering and rehabilitation.

[42] Takahashi K, Groher ME, Michi K-I (1994). Methodology for detecting swallowing sounds. Dysphagia.

[43] Lee J, Sejdić E, Steele CM, Chau T (2010). Effects of liquid stimuli on dual-axis swallowing accelerometry signals in a healthy population. BioMedical Engineering OnLine.

[44] Hamlet S, Penney DG, Formolo J (1994). Stethoscope acoustics and cervical auscultation of swallowing. Dysphagia.

[45] Cichero JA, Murdoch BE (2002). Detection of swallowing sounds: Methodology revisited. Dysphagia.

